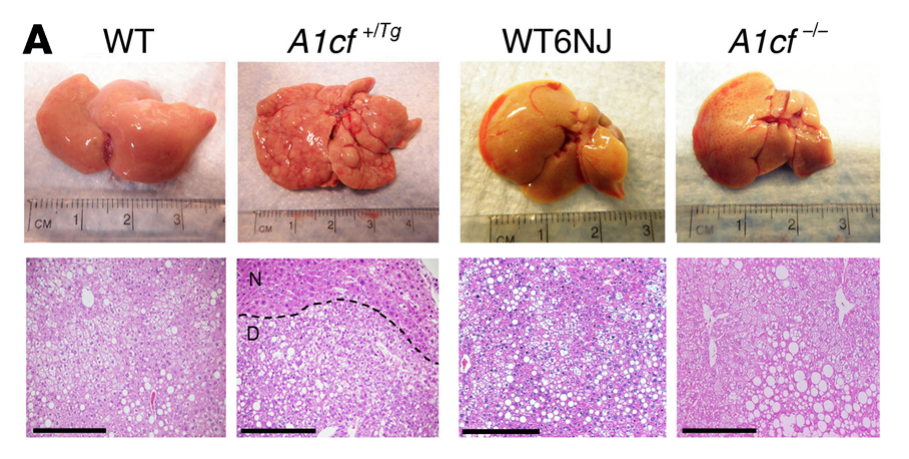# Apobec1 complementation factor overexpression promotes hepatic steatosis, fibrosis, and hepatocellular cancer

**DOI:** 10.1172/JCI194717

**Published:** 2025-06-02

**Authors:** Valerie Blanc, Jesse D. Riordan, Saeed Soleymanjahi, Joseph H. Nadeau, ILKe Nalbantoglu, Yan Xie, Elizabeth A. Molitor, Blair B. Madison, Elizabeth M. Brunt, Jason C. Mills, Deborah C. Rubin, Irene O. Ng, Yeonjung Ha, Lewis R. Roberts, Nicholas O. Davidson

Original citation: *J Clin Invest*. 2021;131(1):e138699. https://doi.org/10.1172/JCI138699

Citation for this corrigendum: *J Clin Invest*. 2025;135(11):e194717. https://doi.org/10.1172/JCI194717

In Figure 7A of the original article, there was an error in the H&E-stained image provided for the WT6NJ sample, which was an inadvertent duplication of the image for the WT sample. The corrected figure, based on the original source data, is provided below. The HTML and PDF versions of the paper have been updated.

The authors regret the error.

## Figures and Tables

**Figure:**